# Gender disparities among prestigious biomedical award recipients in Japan: A cross sectional study

**DOI:** 10.1002/hsr2.70074

**Published:** 2024-09-18

**Authors:** Hayase Hakariya, Tatsuki Ikejiri, Arisa Hakariya, Mayumi Hara

**Affiliations:** ^1^ Laboratory for Human Nature Cultures and Medicine Kyoto Japan; ^2^ Interfaculty Institute of Biochemistry University of Tuebingen Tuebingen Germany; ^3^ Institute for Pharmaceutical and Social Health Sciences Ise Japan; ^4^ Minami Seikyo Hospital Nagoya Aichi Japan

**Keywords:** gender gap, Japan, medical and biomedical awards, unconscious bias, women representation in science

## 1 INTRODUCTION

The limited inclusion of women in medical and biomedical research has been a long‐standing global issue.^1^ Gender and racial inequity continue to pervade contemporary society, including domains such as in leadership positions,^2^, ^3^, ^4^, ^5^ remunerations,^6^ research publication citations,^7^ and professional awards.^8^, ^9^

The gender gap has been observed in the number of original papers,^7^, ^10^ which is an important indicator for evaluating performance, and the proportion of women first author is lower in women scientists.^10^ Moreover, opportunities for women scientists to present and gain recognition at academic conferences have been limited,^11^ and a similar gender gap has also been reported in the chance of obtaining grants.^12^, ^13^ Accumulating these gaps may result in a lower proportion of women in the professional awards.

Previous research investigating the proportion of women among prestigious awards, such as the Nobel Prize,^14^ the Lasker Award,^15^ and other diverse international research awards,^16^ has illuminated the underrepresentation of women within scientific research fields; only 2.7% of Nobel Prize recipients and 7.8% of Lasker Award winners were women. One of the documented reasons for the observed disparities among Nobel Prize winners before 1967 is the lower proportions of women than men among nominees.^14^ However, women's underrepresentation in prestigious awards is presumably caused by a combination of multiple causes including gender discrimination and gender bias.

Historically in Japan, the Basic Law for a Gender‐Equal Society enacted in 1999 has provided milestones and general guidelines for promoting gender equity in society, including academia. Nevertheless, gender gaps still pervade in Japanese higher education,^17^ with women representation in undergraduate 45.6%, in master courses 31.7%, and doctoral courses 34.2%, respectively in 2022.^18^ Remarkably in academia, Japan stood out with strikingly few female academic authors compared with other high‐income countries.^9^ The number of academic papers is one of the important indicators for evaluating scientific performance.^10^ Thus, it is reasonable to hypothesize that there might also be a gender gap in the award conferral stage in Japan. However, few studies have assessed the circumstances surrounding women representation in Japan.

To improve such gender imbalance, Japanese government and universities have begun to promote women's empowerment with relevant policies or practices, emphasizing the importance of diversity, equity, and inclusion, and women in science. For instance, under the 6th Science, Technology, and Innovation Basic Plan, Japan set their milestone to achieve the proportion of women in university up to 23% until 2025.^19^ The Tokyo Institute of Technology is initiating affirmative action for undergraduates, and approximately 14% of student places at the institute are now exclusively available to female applicants.^20^ More recently, the University of Tokyo disclosed its 5‐year scheme to hire 300 women professors and associate professors, aiming to boost the number of female scientists.

Scrutinizing women representation of award recipients in Japan and analyzing if potential inequities have improved after such political/grass‐root practices should be of interest, and this might help unveil specific circumstances that potentially cause a gender gap in Japan. Herein, we investigate the proportion of women among prestigious medical and biomedical award recipients in Japan.

## 2 METHODS

In this cross‐sectional study, we scrutinized three notable Japanese awards in the field of medical and biomedical sciences, each with prestige that warrants speculation regarding potential future Nobel laureates. These awards under scrutiny included the Takeda Prize for Medical Science (established in 1954), the Uehara Prize (established in 1985), and the Keio Medical Science Prize (established in 1996). One reason for choosing these awards is the public availability of the identities of their recipients. The Takeda Prize for Medical Science was chosen because this is one of Japan's most traditional honors with long history. The Keio Medical Science Prize was chosen because the honor represents a contemporary addition, with annual selections comprising one domestic and one international nominee, which made us compare the gender balance between international recipients and domestic recipients. The Uehara Prize was chosen because it was chronologically established in an intermediate of the other two. We excluded notable Japanese awards nominated from diverse fields of humanities, social, and natural sciences to distinguish the potential bias in the medical or biomedical field.

The primary criteria delineated by each foundation are as follows: the Takeda Prize for Medical Science is awarded to researchers with outstanding achievements in basic or clinical fields of medicine; the Uehara Prize is awarded to researchers with outstanding achievements in the life sciences, especially in fields related to health promotion, disease prevention, and treatment; the Keio Medical Science Prize is awarded to researchers with outstanding achievements in medicine and various fields of life science closely related to medicine. The selection processes of recipients for each award were scrutinized from their corresponding websites.

Recipients were identified from the official websites of each respective foundation. Subsequently, four authors independently classified each recipient based on their perceived gender (woman, man, or other), following established methods.^1^, ^15^ Gender categorization is reported in binary (woman or man) in this study, as there were no cases found during our search where recipients' pronounce were as “they” or “hir,” nor did any recipients fall into alternative categories. When categorization was not consistent between authors for some awardees, such discrepancies were resolved through comprehensive discussion between all authors. In addition, we scrutinized the interval between the awardee's terminal degree and award receipt, which we have defined as the “academic age.” The awardee's degrees were investigated through the National Diet Library database.^21^ Statistical analyses were performed with Microsoft Excel^®□^.

## 3 RESULTS

**Figure 1:**
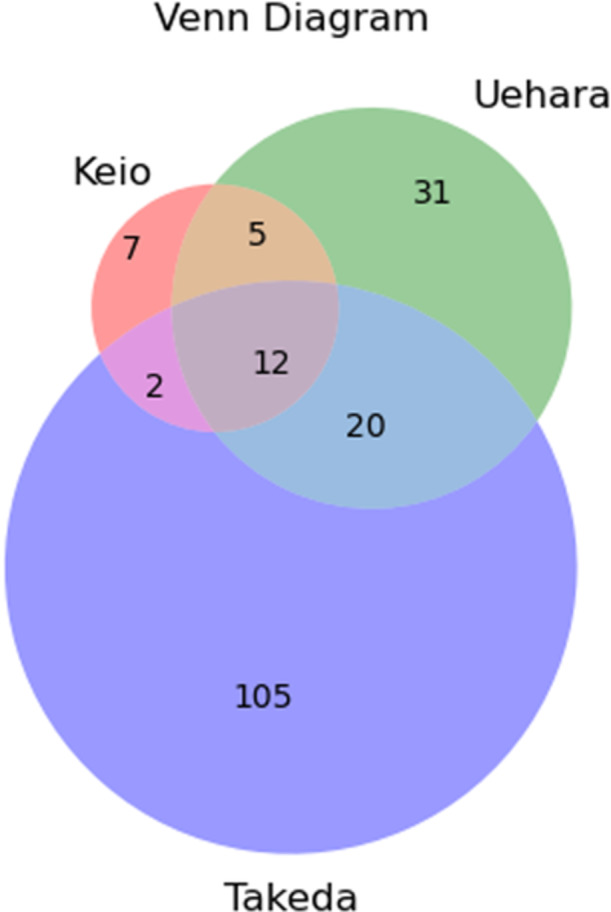
Venn‐diagram of recipients of three awards until 2023. Numbers represent award recipients (*n* = 182). Keio = the Keio Medical Science Prize, Uehara = the Uehara Prize, Takeda = the Takeda Prized for Medical Science.

Two hundred and thirty‐three awards (139 Takeda Prizes for Medical Science, 68 Uehara Prizes, and 26 Keio Medical Science Prizes) have been conferred upon Japanese researchers (Table 1). Among these, 12 recipients won all three awards, while 39 received two, signifying those 182 distinct researchers received at least one (Figure 1). The timeframe between the awardee's terminal degree and award conferral was available for all researchers. The median academic age was 30 years for the Takeda Prize for Medical Science, 26 years for the Uehara Prize, and 28.5 years for the Keio Medical Science Prize awarded to Japanese researchers. Meanwhile, the Keio Medical Science Prizes presented 27 awards to international researchers, with a median academic age of 32 years.

**Table 1:** Winners of prestigious biomedical awards in Japan until 2023.

Award information^a^		Since	Total	Women	Men	Academic age (year)^b^
The Takeda Prize for Medical Science		1954	139	2 (1.4%)	137 (98.6%)	30
Uehara Prize		1985	68	0 (0.0%)	68 (100.0%)	26
Keio Medical Science Prize for Japanese researchers		1996	26	0 (0.0%)	26 (100.0%)	28.5
Keio Medical Science Prize for International researchers		1996	27	3 (11.1%)	24 (88.9%)	32

^a^ As of 15th December 2023.

^b^ Academic age is defined as the time between the awardees' terminal degree and award receipt.

Among Japanese recipients, strikingly, 100% of the Uehara Prize (68/68) and Keio Medical Science Prize recipients (26/26) were men, as were 98.6% (137/139) of the Takeda Prize for Medical Science recipients (Table 1). Among all three awards, only two recipients were women, receiving the Takeda Prize for Medical Science in 2015 and 2023: Kayo Inaba and Yukiko Goto (Table 1). These results substantiate the fact that women award recipients only appeared in the last decade in Japan. Both recipients earned a PhD in science, had a few years of research experience in the United States, and were promoted to professor at the same university where they earned their degrees. Their academic age at the award conferral was 37 years for Kayo Inaba and 31 years for Yukiko Goto, respectively.

On the contrary, 88.9% (24/27) of the Keio Medical Science Prize for international researchers were men and 11.1% (3/27) were women, the three were given in 1999, 2020, and 2021.

The average median academic age between the awardee's terminal degree and award conferral was 28.2 years. Over the last decade (2014 to 2023), 3.8% (2/53) of women comprised the three‐award recipients, which is less than the proportion receiving doctoral degrees 28 years ago in 1995 (15.6%) in Japan.^22^

## 4 DISCUSSION

### 4.1 Findings

We discovered striking gender gap in which the proportion of women receiving any of the three prestigious medical and biomedical science awards remained zero until 2015. Moreover, women comprised only 1.1% (2/182) of scientists who received the prestigious awards，compared to 7.8% (31/397) of Lasker Award recipients,^15^ while academic age of three awards recipients in Japan (Table 1) were comparable to the reported 30 years for Lasker Award recipients.^15^ Considering that 11.1% of the Keio Medical Science Prizes awarded to international researchers were women, there are potentially unique circumstances within the Japanese scientific community that contribute to this stark gender gap. One explanation for the observed gap in this study could be the fact that Japan had fewer female doctoral scientists as it also indicates few female committee members as well as award nominees; In 1995 (28 years ago), only 15.6% of natural science doctoral degrees in Japan were earned by women,^22^ compared to 41.1% in the United States.^23^ However, representation of women was only 3.8% (2/53) among the three‐award recipients over the last decade (2014−2023), which is lower than would be expected based on the proportion earning their doctoral degrees 28 years before in 1995 (15.6%)—and the proportion of women among PhD holders has been increasing over the past decades.^22^ This substantiates a clear gap and simple academic age does not fully account for the observed imbalance.

To fill the gap, it could also be beneficial to make the entire awarding process transparent, from the call for nominations to review, selection, and conferral, as previously recommended.^15^ Moreover, the gender balance between the selection committee, and the award nominees should also be carefully considered to prevent the potential for unconscious bias. In fact, only the Keio Medical Science Prize made the committee member names publicly available among three awards as of 2023 (Table 2). Furthermore, selection committee of the prize in 2021 was marked by an imbalance, comprising twelve men and one woman, demonstrating a clear gender imbalance. This gap was improved up to 25% (12 men and 4 women) in 2023 (Table 2). To prevent potential unconscious biases, this trend should be advocated as an initial step, given that women are not equitably evaluated in research.^24^, ^25^

**Table 2:** Committees and selection processes of Japan's prestigious biomedical awards in 2023.

Award	Committee	Selection process
The Takeda Prize for Medical Science	Only chairperson is open: Ryozo Nagai (male) Other committee information is not available.	Candidates are nominated by executives, councilors, honorary advisors of the foundation as well as previous awardees. The Committee decides on recipients among these candidates. No self‐nomination.
Uehara Prize	Composed of 20−40 members. Individual information is not available	Executives, councilors, honorary executives, advisory boards of the foundation, and presidents of academic societies as well as previous awardees are asked to nominate candidates. The committee select and the board of executives decides final candidates. No self‐nomination.
Keio Medical Science Prize	Publicly available. Composed of 12 males and 4 females.	Candidates are nominated by national and international experts (who are not open). The committee selects the final candidates and the president of the Keio University decides the recipients. No self‐nomination.

The candidates for the three awards were not self‐nominated, indicating that we can rule out the possibility that women were less willing to self‐nominate than men. On the other hand, nominees in the Takeda Prize for Medical Science and the Uehara Prize were recommended also by previous awardees, most of whom are composed of men (Table 2). This might cause potential bias during selection process.

Notably, the only two female Japanese winners were awarded in the last decade: Kayo Inaba in 2015 for her findings in functions of immune response by dendritic cells and Yukiko Goto in 2023 for contributing to identifying the cell‐proliferation signal MAP kinase and related factors. These recent wins may reflect a positive trend in promoting women scientists in Japan, possibly influenced by movements such as the one led by the Japan Science and Technology Agency.^26^ However, further drastic changes and efforts are warranted among scientific communities, the general public, and policymakers to address this severe gender imbalance.

Moreover, it might be noteworthy that we found another possible issue regarding diversity and inclusion: winners of the Takeda Prize for Medical Science were all Japanese, despite the eligibility criteria clearly stating that individuals of any nationality can be recipients. The fact might indicate that people of other nationalities were not chosen due to the unconscious bias, even though Japan has a small proportion of foreign residents (2.2% as of 2020).^27^

### 4.2 Policy and social implications

At the very least, further studies that could unveil at which stages are there problems with gender differences and requires intervention to solve the issue are warranted: learning opportunities leading up to university admission, degree acquisition, research positions, research funding acquisition, publication, reviewers involved in providing these opportunities.

Nonetheless, step by step, Japan should start rectifying the gender gap (and potential gender bias) in each academic career level, given the already existing discrepancy at the student doctoral stage.^28^ First, initiatives should aim to increase the number of women studying at universities. Simultaneously, we also note that simply filling numbers is insufficient for increasing internationally viable personnel to be conferred prestigious awards. Reducing obstacles unique to women's career development, such as pregnancy and childbirth, beyond the issue of gender gaps, is essential. Indeed, facilities or researchers' work styles should also be reconsidered, given that they sometimes have to monitor their samples the day long in the medical or biomedical field.

## 5 LIMITATIONS

Our investigation had limitations. First, the anonymity of the nomination process for each award precluded us from rating whether women were equally nominated at panels. Second is the potential misclassification of gender by researchers. On the other hand, the authors also note that gender perceptions of the recipients by third parties are important, as it is susceptible to bias.^29^ Additionally, our methods were reported previously.^1^, ^15^ Third, the extreme gender gap we observed in the medical/biomedical field is not necessarily generalizable to the other scientific fields. The gap has been academically reported to vary among fields.^28^, ^30^, ^31^ Therefore, careful interpretation is required for our study. Lastly, our investigation did not reveal the causation of the gender gap at the award conferral stage. Clarifying at which stage is there the gender gap problem should be indispensable to address the issue.

## AUTHOR CONTRIBUTIONS

**Hayase Hakariya**: Conceptualization; investigation; formal analysis; writing—original draft; writing—review and editing; supervision; data curation; methodology; visualization; resources; validation. **Tatsuki Ikejiri**: Investigation; writing—review and editing; data curation. **Arisa Hakariya**: Investigation; writing—review and editing; data curation. **Mayumi Hara**: Investigation; writing—review and editing; data curation.

## CONFLICT OF INTEREST STATEMENT

The authors declare no conflict of interest.

## ETHICS STATEMENT

All information was publicly available and ethics approval was not required. Data were collected from June 21 to August 17, 2023, and updated on December 15, 2023. This cross‐sectional study was performed only with publicly available data, and no patients or individual patient record were involved. Therefore, no institutional review board approval and patient's informed consent were necessary.

## TRANSPARENCY STATEMENT

The lead author Hayase Hakariya affirms that this manuscript is an honest, accurate, and transparent account of the study being reported; that no important aspects of the study have been omitted; and that any discrepancies from the study as planned (and, if relevant, registered) have been explained.

## Data Availability

The data that support the findings of this study are available from the corresponding author upon reasonable request.
